# Proteins involved in the endoplasmic reticulum stress are modulated in synovitis of osteoarthritis, chronic pyrophosphate arthropathy and rheumatoid arthritis, and correlate with the histological inflammatory score

**DOI:** 10.1038/s41598-020-70803-7

**Published:** 2020-09-04

**Authors:** Dominique de Seny, Elettra Bianchi, Dominique Baiwir, Gaël Cobraiville, Charlotte Collin, Mégane Deliège, Marie-Joëlle Kaiser, Gabriel Mazzucchelli, Jean-Philippe Hauzeur, Philippe Delvenne, Michel G. Malaise

**Affiliations:** 1 Laboratory of Rheumatology, GIGA Research, CHU Liege, Tour GIGA, +2, 4000 Liege, Belgium; 2 Department of Pathology, GIGA Research, CHU Liege, 4000 Liège, Belgium; 3 GIGA Proteomics Facility, University of Liege, 4000 Liege, Belgium; 4 Mass Spectrometry Laboratory, MolSys Unit Research, University of Liege, 4000 Liege, Belgium

**Keywords:** Rheumatology, Rheumatic diseases

## Abstract

It is now well recognized that osteoarthritis (OA) synovial membrane presents inflammatory components. The aim of this work is to provide evidence that similar inflammatory mechanisms exist in synovial membrane (n = 24) obtained from three pathologies presenting altogether an inflammatory gradient: OA, chronic pyrophosphate arthropathy (CPPA) and rheumatoid arthritis (RA). Synovial biopsies were first characterized by a histological score based on synovial hyperplasia and infiltration of lymphocytes, plasma cells, polymorphonuclear and macrophages. All biopsies were also analyzed by 2D-nano-UPLC-ESI-Q-Orbitrap for protein identification and quantification. Protein levels were correlated with the histological score. Histological score was in the range of 3 to 8 for OA, 5 to 13 for CPPA and 12 to 17 for RA. Of the 4,336 proteins identified by mass spectrometry, 51 proteins were selected for their strong correlation (p < 0.001) with the histological score of which 11 proteins (*DNAJB11, CALR, ERP29, GANAB, HSP90B1, HSPA1A, HSPA5, HYOU1, LMAN1, PDIA4,* and *TXNDC5*) were involved in the endoplasmic reticulum (ER) stress. Protein levels of S100A8 and S100A9 were significantly higher in RA compared to OA (for both) or to CPPA (for S100A8 only) and also significantly correlated with the histological score. Eighteen complement component proteins were identified, but only *C1QB* and *C1QBP* were weakly correlated with the histological score. This study highlights the inflammatory gradient existing between OA, CPPA and RA synovitis either at the protein level or at the histological level. Inflamed synovitis was characterized by the overexpression of ER stress proteins.

## Introduction

Osteoarthritis (OA) was for long considered as a degenerative cartilage disease for which synovitis was only visualized in the late stages and considered as secondary to mechanic aggression of bone and cartilage degradation. However, several observations demonstrated that synovitis could appear even in the early stages of OA. Synovium can also acquire an “inflammatory” phenotype in OA with similar characteristics than those observed in rheumatoid arthritis (RA) for which synovitis is the hallmark: [i.e. synovial lining and villous hyperplasia, infiltration by macrophages and lymphocytes, neo-angiogenesis and fibrosis]^1,2^. Using magnetic resonance imaging (MRI), Roemer et al*.* noted a synovitis in 96.3% of knee joints with effusion and in 70% of knee joint without effusion^3^. We previously published by using ultrasonography (US) examination that 53.7% (322/600) of patients with painful knee OA had no sign of inflammation whereas 2.7% (16/600) had synovitis alone, 14.2% (85/600) had both synovitis and effusion and 29.5% (177/600) had joint effusion alone^4^. US knee synovitis and US joint effusion were significantly associated with a more severe radiological grade (Kellgren–Lawrence grade ≥ 3) and a moderate-important joint effusion at clinical examination^4^. Further, several other studies have confirmed the correlation between synovitis area observed by MRI and specific histologic features of synovitis^5^.

Two major pathways at least can explain the development of synovitis: activation of toll-like receptors (TLR) and activation of the complement cascade^1^. Endogenous “damage-associated molecular patterns” (DAMPS) can activate the innate immune response through TLRs recognition promoting pro-inflammatory mediators secretion^6,7^.

Activation of the complement cascade induces complement deposits sparsely found in the synovium of OA patients. Deposits of synovial complement components were only observed during acute OA flare but not during chronic OA^8^. More recently, proteomic analyses of OA synovial fluids^9,10^ and transcriptomic studies of OA synovial membranes^10^ confirmed the expression and activation of complement in joints^11^.

Proteomic analysis of synovial tissue was rarely performed^12,13^ and none was compared to the histological pattern of synovium. In this work, we compared protein profiles generated by a proteomic study to the histological features of synovial biopsies obtained from patients with OA, chronic pyrophosphate arthropathy (CPPA) or RA. We have highlighted that OA synovitis share at a lower degree many common features with RA synovitis including abnormal synoviocytes proliferation and infiltration of lymphocytes, plasma cells and macrophages. CPPA is caused by the deposition of calcium pyrophosphate crystals in the joint tissues triggering inflammation and inducing synovitis similarly to OA and RA. This study also unraveled an increased gradient of inflammation and synovial lining hyperplasia among the three pathologies both at the protein and histological levels. Finally, inflamed synovitis was characterized by the overexpression of endoplasmic reticulum (ER) stress proteins that have never been described for most of them in OA synovium and that could play a role in cytokines production and fibroblasts proliferation.

## Methods

### Patients and synovial tissue

All experiments undertaken with patient material complied with the regulations and ethical guidelines of the CHU of Liege, Belgium and were approved by the CHU ethical committee (B707201732662; ref: 2017/147). Informed consent was obtained from all subjects. Synovial biopsies of OA (n = 9), CPPA (n = 7) and RA patients (n = 8) were obtained by needle arthroscopy from affected knees. For each patient, three synovial fragments were stored at − 80 °C until used for proteomic study. Three other fragments were also processed for formalin fixation (24 h) using a standard procedure and were embedded in paraffin for microscopic examination of the hematoxylin and eosin (H&E) stained sections and histological scoring.

### Study design

All samples in each disease group were selected retrospectively from a cohort including 137 synovitis biopsies after clinical examination of patients, biological analysis of serology and histological characterization of synovitis by rheumatologists and pathologists. Following criteria were applied to choose the 24 biopsies: (1) biopsies were provided from untreated patients who did not receive any corticoids or any modifying anti-rheumatic drugs (including biologics), (2) biopsies illustrated all together an inflammatory gradient according to histological characterization.

### Histological inflammatory score

Hematoxylin eosin stained sections were scored as previously done for routine clinical analysis and randomly analyzed as described in Tak et al.^14^. Briefly, all areas of each biopsy section were examined by trained anatomopathologists (P.D. and E. B.). Histological features included synovial hyperplasia and the degree of infiltration of lymphocytes, plasma cells or polymorphonuclear cells (PMN), separately. The TAK score^14^ was slightly modified: (a) synovial hyperplasia was scored on a five-point scale (0–4) instead of a four-point scale (0–3) because synovitis hallmark in RA as in OA is proliferation and hyperplasia of the lining cells^2^ and (b) PMN infiltration was scored on a four-point scale (0–3) instead of a five-point scale (0–4) because PMN infiltration is less intense than any other mononuclear cells infiltration. Macrophage infiltration was also determined by using immunohistochemistry with an anti-CD68 antibody [anti-CD68 (KP-1) Primary Antibody, Ventana Medical System], and the CD68 expression was scored separately using a semi-quantitative 4 scale score (0–3) with 0 for no infiltration, 1, 2 and 3 for respectively mild, moderate and severe infiltration using a pre-defined atlas^15^. The histological inflammatory score was determined by the sum of the components, as for other methods^14,15^, leading to a maximum of 18.

### LC–MS/MS analysis for proteomic analysis

The biopsies were weighted (5 mg) and resuspended in 300 µL of RIPA buffer in the presence of complete and phospho stop solutions. The biopsies were then disrupted using a bi-switch (+ 60 mg of μbeads) for cycles of 30 s high speed and 30 s low speed at 4 °C, during 2 × 15 min, to allow proteins dissolution in the buffer. The protein concentration of each sample was determined using the RCDC protein assay kit according to manufacturer recommendations (BioRad, Hercules, CA, USA). For each sample, 15 μg of protein were diluted in ammonium bicarbonate (50 mM) to get a protein concentration of 0.5 μg/μL. The proteins were then reduced (DTT), alkylated with iodoacetamide and precipitated using the 2D clean-up kit (GE Healthcare, Belgium). The protein pellets were then resuspended in ammonium bicarbonate (50 mM) at a concentration of 0.5 μg/μL and digested with trypsin. For each sample, 3.5 μg of peptides were desalted using Ziptip C18 (Millipore Corp., Billerica, MA, USA) according to the manufacturer's instructions. The eluted fractions were then dried by speed-vac. Dry pellets were stored at − 20 °C until used for analysis. Before injection into the 2D-nano-UPLC system, 2.5 μg of the digested proteins were resuspended in 9 μL of 100 mM ammonium formate solution adjusted to pH10. A standard MassPREP (MPDS mixture) digestion mixture (Waters Corp., Milford, USA) which contains a mixture of yeast enolase (ENO1, P00924), rabbit glycogen phosphorylase b (GPB, P00489), yeast alcohol dehydrogenase (ADH, P00330) and bovine serum albumin (BSA, P02769) was spiked in each sample at a quantity of 150 fmoles of ADH digest per injection.

All samples were injected on a 2D-nanoAquity UPLC (Waters, Corp., Milford, USA) coupled online with a ESI-Q-Orbitrap (Q Exactive, Thermo Fisher Scientific) in positive ion mode, as previously described^16^. Briefly, the liquid chromatography approach used was a two-dimensional method (2D-LC) comprising three steps of 180 min. The three steps were carried out on a high pH column with increasing percentage of acetonitrile. The eluted peptides were then injected onto a low pH column for which each step consists of a 5 min gradient from 99% buffer A (A = H_2_O, 0.1% formic acid, B = acetonitrile) to 93% of A followed by a gradient of 135 min from 93% of A to 65% of A. The acquisition method was a TopN-MSMS where N was set to 12, meaning that the spectrometer acquired one Full MS spectrum, selected the 12 most intense peaks in this spectrum (singly charged precursors excluded) and recorded a Full MS2 spectrum of each of these 12 compounds. The parameters for MS spectrum acquisition were: mass range from 400 to 1,750 *m/z*, resolution of 70,000, automated gain control (AGC) target of 10^6^ or maximum injection time of 200 ms. The parameters for MS2 spectrum acquisition were: isolation window of 2.0 *m/z*, collision energy (NCE) of 25, resolution of 17,500, AGC target of 10^5^ or maximum injection time of 50 ms. The database searches were performed by the software MaxQuant ver.1.5.2.8. Protein identifications were considered as significant if a protein was identified with at least two peptides including at least one unique peptide. The false discovery rate (FDR) both at the Peptide Spectrum Match (PSM) and at the protein levels was set at 0.01 (1%) in MaxQuant.

### Data analysis

Epidemiological data of the three groups (OA, CPPA and RA) were compared using the Kruskal Wallis test with the posthoc test of Dunn’s for continuous variables and Chi square test for qualitative variables. Comparison of two-by-two groups for K&L was performed with Mann Whitney test.

Maxquant analysis leads to the identification of peptides based on MS/MS spectra and the quantification of protein is done based on MS1 intensities of peptides that are then normalized using LFQ algorithm in MaxQuant^17^. The label free quantification (LFQ) intensities can be directly compared between samples (for a given protein) and can be imported in Perseus software (version 1.5.5.0) for statistical differential analysis. Only proteins identified and quantified in seven biopsies of at least one of the three groups (OA, CPPA and RA) were considered for further analysis. 1871 proteins were selected accordingly. LFQ intensities were Log2 transformed for all statistical analyses. Correlation coefficients were obtained using Pearson test after verifying that all values passed the D’Agostino normality test. Statistical significance test was applied to all correlation parameters of Tables 1 and 2. Calculated t-values were higher compared to theoretical critical values for all correlation parameters of Tables 1 and 2, and therefore confirm the linear relationship (correlation) between histological score (x) and protein intensities (y) at the 0.05 α level.

**Table 1:** Patients description.

	OA	CPPA	RA	P value
n	9	7	8	
Age [median (interval)]	55 (36–89)	65 (50–74)	57 (29–78)	0.3
% of woman	88% (8/9)	71% (5/7)	62% (5/8)	0.44
BMI [median (interval)]	32.2 (17.6–41.9)	24.2 (22–33)	24.4 (16.4–33.9)	0.4
K&L score [median (interval)]	3 (0–4)	2 (0–4)	–	0.6
Histological inflammatory score	4 (3–8)	5 (5–13)	14 (12–17)	0.0003
Anti-CCP (positive %)	0%	0%	60%	0.002
Rheumatoid factor (positive %)	0%	0%	40%	0.032
CRP (positive %)	20%	40%	90%	0.020
ESR (positive %)	10%	0%	60%	0.01

Clinical and pathological characteristics of patients with osteoarthritis (OA), chronic pyrophosphate arthropathy (CPPA) or rheumatoid arthritis (RA).

*OA* osteoarthritis, *CPPA* chronic pyrophosphate arthropathy, *RA* rheumatoid arthritis, *BMI* body mass index, *K&L* Kellgren and Lauwrence score, *anti-CCP* anti-cyclic citrullinated peptide, *CRP* C reactive protein, *ESR* erythrocyte sedimentation rate

**Table 2:** Correlation parameters between protein intensities and histological inflammatory score.

Gene ID	Prot ID	Prot name	n	r	P-value
*LSP1*	P33241	Lymphocyte-specific protein 1	16	0.83	< 0.0001
*MZB1*	Q8WU39	Marginal zone B- and B1-cell-specific protein	15	0.80	0.0004
*MANF*	P55145	Mesencephalic astrocyte-derived neurotrophic factor	23	0.79	< 0.0001
*EML4*	Q9HC35	Echinoderm microtubule-associated protein-like 4	20	0.78	< 0.0001
*LAP3*	P28838	Cytosol aminopeptidase	24	0.77	< 0.0001
*DNAJB11*	Q9UBS4	DnaJ homolog subfamily B member 11	24	0.77	< 0.0001
*DEFA1*	P59665	Neutrophil defensin 1	21	0.76	< 0.0001
*ERP29*	P30040	Endoplasmic reticulum resident protein 29	24	0.75	< 0.0001
*IDH2*	P48735	Isocitrate dehydrogenase [NADP], mitochondrial	24	0.75	< 0.0001
*LCP1*	P13796	Plastin-2	24	0.74	< 0.0001
*TXNDC5*	Q8NBS9	Thioredoxin domain-containing protein 5	24	0.73	< 0.0001
*HSP90B1*	P14625	Endoplasmin or glucose-related protein 94 (GRP94)	24	0.73	< 0.0001
*CALR*	P27797	Calreticulin	24	0.73	< 0.0001
*PRDX4*	Q13162	Peroxiredoxin-4	24	0.73	< 0.0001
*SRP72*	O76094	Signal recognition particle subunit SRP72	22	0.73	0.0001
*HSPA5*	P11021	78 kDa glucose-regulated protein (GRP78) or BiP	24	0.72	< 0.0001
*ARHGDIB*	P52566	Rho GDP-dissociation inhibitor 2	24	0.72	< 0.0001
*PDIA4*	P13667	Protein disulfide-isomerase A4	24	0.72	< 0.0001
*TAPBP*	O15533	Tapasin	18	0.72	0.0009
*CORO1A*	P31146	Coronin-1A	24	0.71	< 0.0001
*S100A8*	P05109	Protein S100-A8	24	0.71	0.0001
*CTSS*	P25774	Cathepsin S	22	0.69	0.0003
*CTSZ*	Q9UBR2	Cathepsin Z	24	0.69	0.0002
*MNDA*	P41218	Myeloid cell nuclear differentiation antigen	23	0.69	0.0003
*LMNB1*	P20700	Lamin-B1	24	0.69	0.0002
*TUBA4A*	P68366	Tubulin alpha-4A chain	24	0.68	0.0002
*PMM2*	O15305	Phosphomannomutase 2	20	0.68	0.0009
*CNPY2*	Q9Y2B0	Protein canopy homolog 2	24	0.68	0.0003
*PTPRC*	P08575	Receptor-type tyrosine-protein phosphatase C	23	0.68	0.0004
*S100A9*	P06702	Protein S100-A9	24	0.68	0.0003
*LMAN1*	P49257	Protein ERGIC-53	24	0.68	0.0003
*EEF1G*	P26641	Elongation factor 1-gamma	24	0.67	0.0004
*STAT1*	P42224	Signal transducer and activator of transcription 1-alpha/beta	23	0.66	0.0007
*GBP1*	P32455	Interferon-induced guanylate-binding protein 1	24	0.65	0.0005
*GNB2L1*	P63244	Guanine nucleotide-binding protein subunit beta-2-like 1	24	0.65	0.0006
*PARP1*	P09874	Poly [ADP-ribose] polymerase 1	24	0.64	0.0007
*PFN1*	P07737	Profilin-1	24	0.64	0.0008
*HYOU1*	Q9Y4L1	Hypoxia up-regulated protein 1 (GRP170)	24	0.63	0.0009
*GANAB*	Q14697	Neutral alpha-glucosidase AB	24	0.63	0.001
*TNXB*	P22105	Tenascin-X	23	− 0.65	0.0008
*CRTAC1*	Q9NQ79	Cartilage acidic protein 1	22	− 0.66	0.0009
*HSPA1A*	P0DMV8	Heat shock 70 kDa protein 1A/1B (HSP70-1)	24	− 0.66	0.0005
*SPTBN1*	Q01082	Spectrin beta chain. non-erythrocytic 1	24	− 0.67	0.0003
*SNTB2*	Q13425	Beta-2-syntrophin	24	− 0.67	0.0004
*LEMD2*	Q8NC56	LEM domain-containing protein 2	21	− 0.67	0.0008
*LMNB2*	Q03252	Lamin-B2	24	− 0.68	0.0003
*CKB*	P12277	Creatine kinase B-type	20	− 0.68	0.0009
*GPX3*	P22352	Glutathione peroxidase 3	23	− 0.69	0.0002
*CPQ*	Q9Y646	Carboxypeptidase Q	18	− 0.75	0.0004
*SYNE3*	Q6ZMZ3	Nesprin-3	23	− 0.75	< 0.0001
*SCARA5*	Q6ZMJ2	Scavenger receptor class A member 5	15	− 0.85	< 0.0001

Intensities of 51 proteins quantified by MS/MS were significantly correlated to the histological score. n = number of biopsies for which the protein was detected; r = coefficient correlation (Pearson test). P-values are statistically significant < 0.001.

One-way ANOVA test with Tukey’s multiple comparison test was used for comparing protein intensities between the three disease groups [OA, CPPA and RA].

DAVID Bioinformatics resources 6.8 was used for the identification of KEGG pathways. STRING 10.5 was used for functional protein association network.

### Ethics approval and consent to participate

All experiments undertaken with patient material complied with the regulations and ethical guidelines of the CHU of Liege, Belgium.

## Results

### Characteristics of patients

Clinical and biologic data are summarized in Table 1. Parameters related to age, gender and BMI were not statistically different between OA, CPPA and RA patients. Severity of OA and CPPA was defined according to the Kellgren Lauwrence (K&L) grade^18^ and was not statistically different between the two groups. Histological inflammatory score was significantly different between the three groups (P value = 0.0003) and was higher in RA compared to OA (P < 0.001) or CPPA (P < 0.05) but not different between OA and CPPA groups according to the posthoc test. CRP values exceeding the normal range were observed in 20%, 40% and 90% of OA, CPPA and RA patients, respectively; all patients being untreated by corticosteroids or any disease modifying anti-rheumatic drugs, including biologics.

### Histological inflammatory score

**Figure 1:**
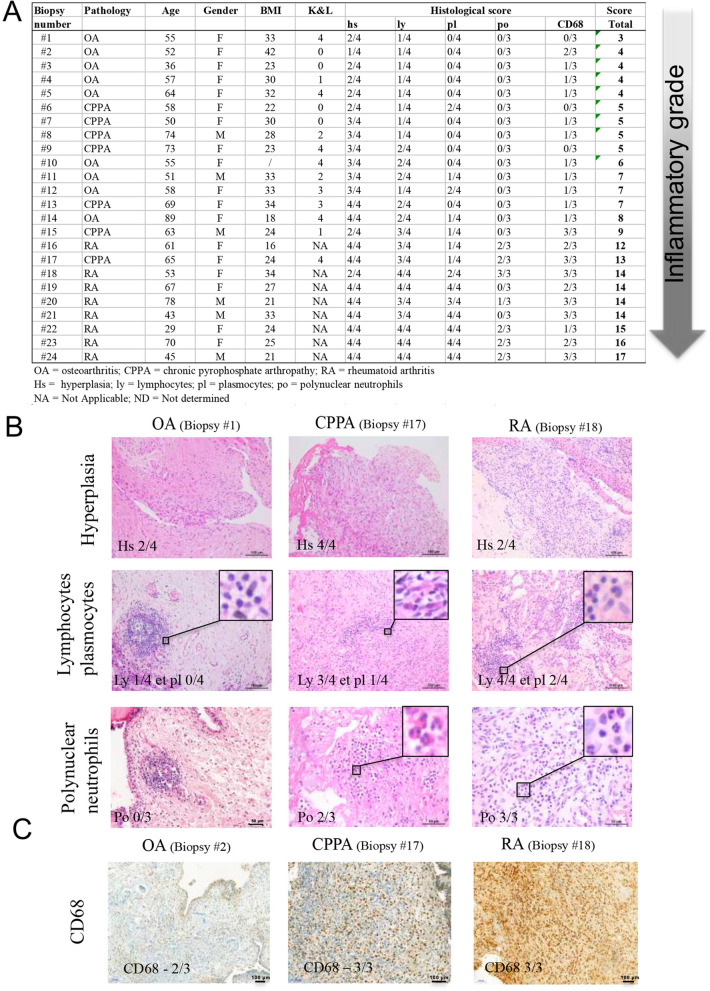
Histological scoring of synovial biopsies. **(A)** Classification of OA, CPPA and RA synovial biopsies (n = 24) according to the histological inflammatory score based on the following criteria: synovial hyperplasia (hs, 0–4), infiltration of lymphocytes (ly, 0–4), plasmocytes (pl, 0–4), polynuclear neutrophils (po, 0–3) and macrophages (CD68, 0–3). **(B)** Histological representation of hematoxylin eosin stained sections for synovial hyperplasia and infiltration of lymphocytes/plasmocytes and polynuclear neutrophils in one OA, CPPA or RA biopsy. **(C)** Immunohistochemistry using anti-CD68 antibody showing macrophage infiltration in OA, CPPA and RA synovial biopsies.

Histological inflammatory score was in the range of 3 to 8 for OA, 5 to 13 for CPPA and 12 to 17 for RA, which represents a continuum for the inflammatory process of the synovial membrane through the 24 biopsies, the least severe being the OA group in contrast to CPPA (medium score) and RA (highest score) (Table 1)*.* Histology scoring for all patients is represented in Fig. 1A. Of note, there is an overlap between the 3 pathologies, and some patients with OA have already a medium high inflammatory score*.* Synovial hyperplasia and infiltration of lymphocytes were observed in all samples (Fig. 1A, B), whereas plasmocytic infiltration was observed in only 3/9 OA (33%), 3/7 (43%) CPPA and 8/8 RA synovitis, and PMN infiltration in 0/9 OA, in 1/7 (14%) CPPA and in 6/8 (75%) RA synovitis (Fig. 1A, B). Macrophage infiltration was present in 8/9 (89%) of OA, in 5/7 (71%) of CPPA and in 8/8 RA synovitis (Fig. 1A, C). Interestingly, macrophage score (CD68) was correlated with CRP levels for the 24 biopsies and a significant correlation factor of 0.66 (P value = 0.0063) was obtained. CRP levels were also highly correlated with lymphocytes infiltration (r = 0.73; P value = 0.0022).

### Proteomics analysis

**Figure 2:**
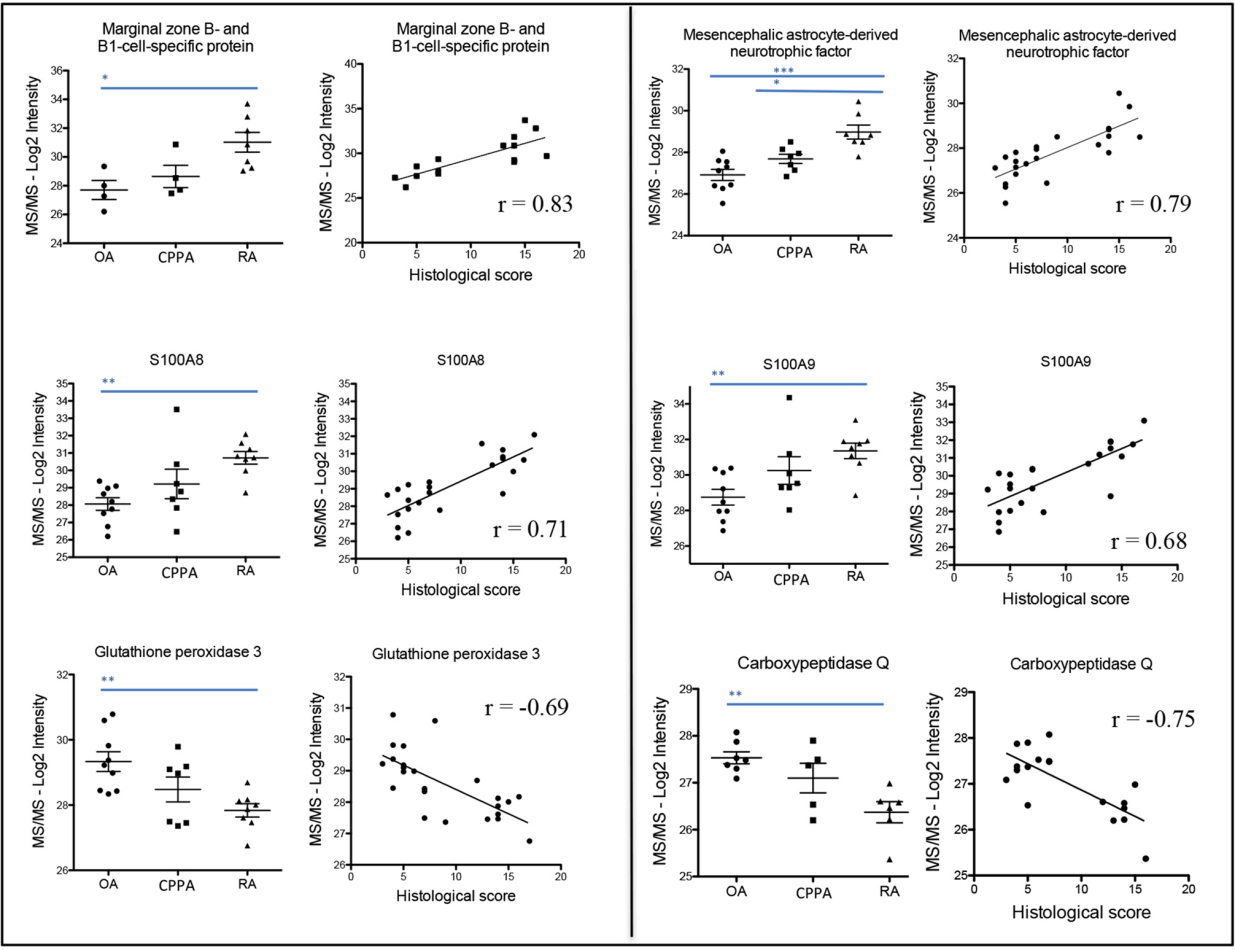
Distribution of protein intensities among the 3 groups (OA, CPPA and RA) and correlation with the histological inflammatory score. Illustration of some proteins from Table 2 for which log2 intensities obtained by MS/MS are represented among the three groups (OA, CPPA and RA) and statistically correlated to the histological inflammatory score. *, **, *** Represent P-values < 0.05; 0.01 and 0.001, respectively.

Proteins were extracted and digested from the 24 biopsies for proteomic analysis by mass spectrometry (MS/MS). 4336 proteins were identified by MS/MS, but only 1871 proteins were selected for their significant identification in seven biopsies of at least one of the three groups (OA, CPPA or RA). The 1871 proteins intensities were then correlated with their corresponding histological inflammatory score. Fifty-one proteins presented a statistically significant correlation with a P value being at least < 0.001 (Table 2), including 39 proteins with a positive correlation (r > 0.63) and 12 proteins with a negative correlation (r < − 0.65) (Table 2). Some proteins of Table 2 are illustrated in Fig. 2 for the three disease groups, as well as correlation parameters between MS/MS log2 protein intensities and respective histological inflammatory score. In Table 2, there were 31 proteins detected in the entire set of the 24 biopsies, including 27 proteins being positively correlated with the histological inflammatory score and 4 proteins being negatively correlated (Table 2).

We also confirmed that DAMP proteins, S100A8 and S100A9, were detected in the 24 synovial membrane biopsies and that their protein levels were strongly increased in RA compared to OA biopsies (ANOVA, Tuckey post hoc test P < 0.01). Only S100A8 but not S100A9 protein levels were discriminant between CPPA and RA groups (P < 0.01). As expected, a strong correlation between S100A8 and S100A9 protein levels (r = 0.95, P < 0.0001) was determined among the three groups.

**Figure 3:**
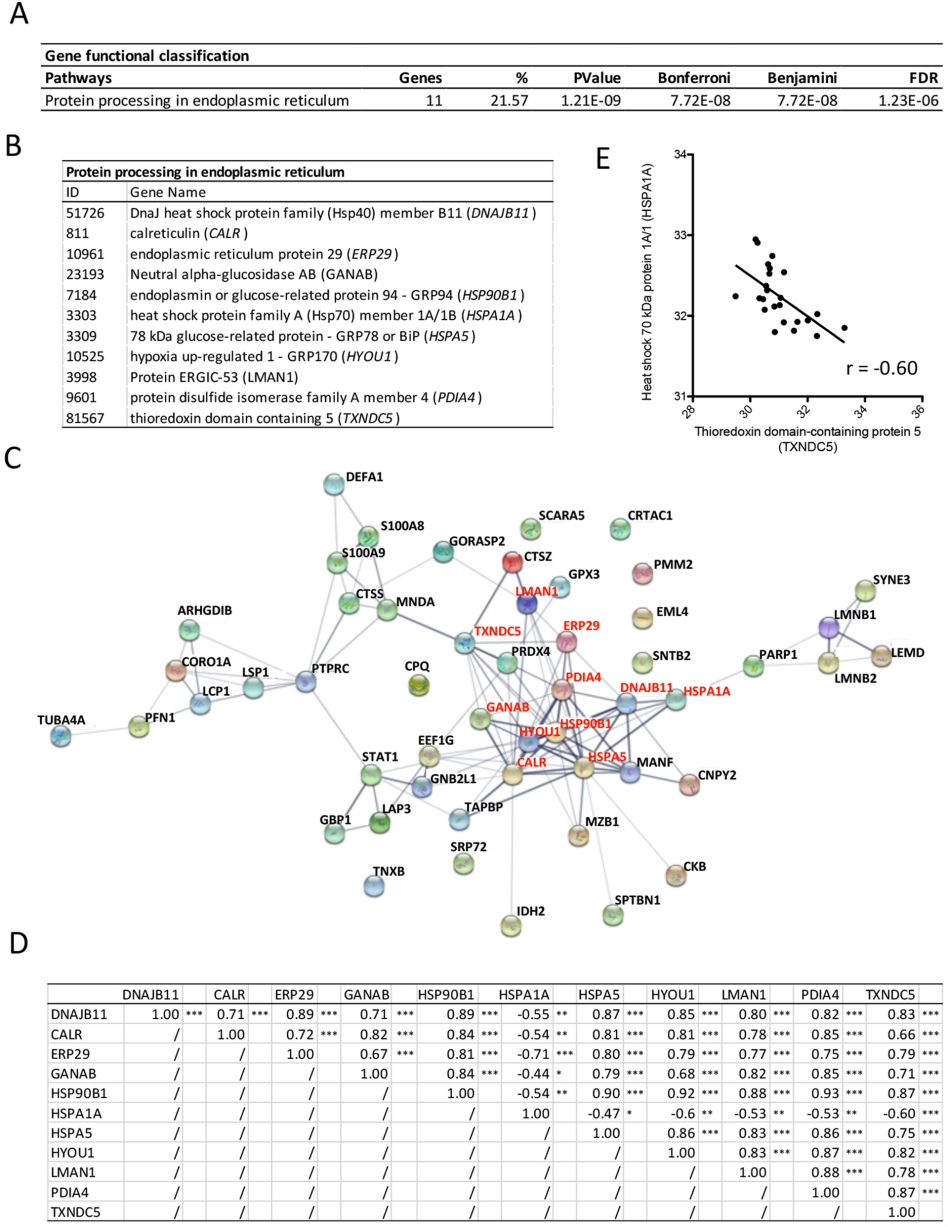
ER stress proteins detected in the inflamed synovial membrane: **(A)** DAVID analysis performed on the 51 proteins highlighted for their significant correlation to the histological inflammatory score, for their functional classifications. The pathway entitled “protein processing in endoplasmic reticulum (ER)” was selected. **(B)** Proteins involved in the ER pathway according to DAVID analysis. **(C)** STRING protein–protein interaction among the 51 proteins highlighted in Table 2. Red writings indicate proteins involved in the ER network according to DAVID analysis. **(D)** Correlation parameters between the 11 proteins involved in the ER according to DAVID. **(E)** Negative correlation between HSPA1A and TXNDC5 protein levels.

**Figure 4:**
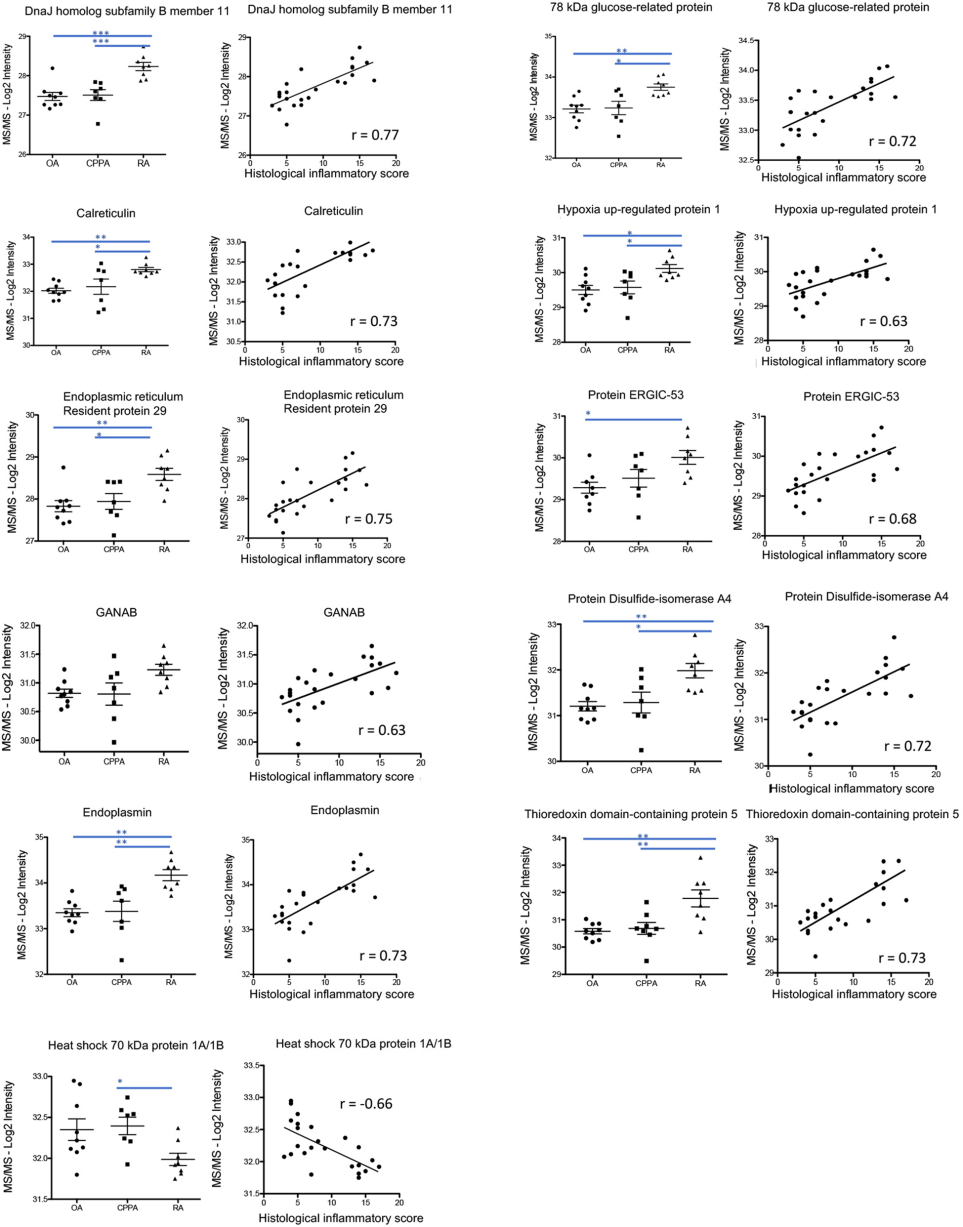
Distribution of ER protein intensities among the 3 groups (OA, CPPA and RA) and correlation with the histological inflammatory score. Illustration of the 11 ER proteins for which log2 intensities obtained by MS/MS are represented among the three groups (OA, CPPA and RA) and statistically correlated to the histological inflammatory score. *, **, ***Represent P-values < 0.05; 0.01 and 0.001, respectively.

DAVID analysis was performed on the 51 proteins to highlight their functional classifications (Fig. 3A). The pathway entitled “protein processing in endoplasmic reticulum (ER)” was selected and weighted in the balance for 22% (Fig. 3A). In this pathway, 11 proteins were identified: *DNAJB11*, *CALR*, *ERP29*, *GANAB, HSP90B1*, *HSPA1A*, *HSPA5*, *HYOU1*, *LMAN1*, *PDIA4*, and *TXNDC5* (Fig. 3B) and were connected by the STRING protein–protein interaction network (see red writings in Fig. 3C). Statistically significant positive correlations were confirmed and summarized in Fig. 3D, except for *HSPA1A* that was negatively correlated to the 10 others. Out of that observation, we calculated the ratio of *HSPA1A* to *TXNDC5* protein levels that was negatively correlated (r = − 0.6, P = 0.002) with the histological inflammatory score (Fig. 3E). The 11 ER protein levels were also illustrated in Fig. 4 for the three disease groups, as well as correlation parameters between MS/MS log2 protein intensities and respective histological inflammatory score (Fig. 4).

Although DAVID analysis did not highlight a pathway centered on the alarmins S100A8 and S100A9, both proteins were significantly correlated with almost all 50 other proteins selected in Table 2, except for *PARP1* and *CRTAC1* for S100A8 or S100A9, and *GANAB* and *GNB2L1* for S100A9 (Table 3). The same 37 proteins as in Table 2 were positively correlated with S100A8 or S100A9, as well as the 12 proteins that were negatively correlated (Table 3).

**Table 3:** Correlation parameters between S100A8/S100A9 and the 49 protein intensities correlated to the histological inflammatory score.

	S100A8		S100A9	
	Pearson r	P value	Pearson r	P value
Protein S100A8/A9	0.9545	***	0.9545	***
Neutrophil defensin	0.9131	***	0.8637	***
Lymphocyte-specific protein 1	0.8527	***	0.8197	***
Coronin-1A	0.8417	***	0.8041	***
Plastin-2	0.837	***	0.8021	***
Cathepsin Z	0.8266	***	0.7937	***
Myeloid cell nuclear differentiation antigen	0.8118	***	0.7771	***
Tapasin	0.7384	***	0.6698	**
Profilin-1	0.7348	***	0.6748	***
Mesencephalic astrocyte-derived neurotrophic factor	0.7223	***	0.7417	***
Cathepsin S	0.7125	***	0.6177	**
Receptor-type tyrosine-protein phosphatase C	0.707	***	0.643	***
Rho GDP-dissociation inhibitor 2	0.6958	***	0.6217	**
Calreticulin	0.68	***	0.6088	**
Cytosol aminopeptidase	0.679	***	0.6264	**
Marginal zone B- and B1-cell-specific protein	0.667	**	0.7098	**
Elongation factor 1-gamma	0.6522	***	0.5827	**
Lamin-B1	0.6505	***	0.587	**
Echinoderm microtubule-associated protein-like 4	0.6346	**	0.5933	**
Phosphomannomutase 2	0.614	**	0.6091	**
Endoplasmic reticulum resident protein 29	0.5962	**	0.644	***
Peroxiredoxin-4	0.578	**	0.5787	**
Protein canopy homolog 2	0.5758	**	0.6744	***
Signal transducer and activator of transcription 1-alpha/beta	0.5704	**	0.5427	**
DnaJ homolog subfamily B member 11	0.5504	**	0.5635	**
Interferon-induced guanylate-binding protein 1	0.5373	**	0.566	**
Hypoxia up-regulated protein 1 (GRP170)	0.5357	**	0.5298	**
78 kDa glucose-regulated protein (GRP78) or BiP	0.5233	**	0.5043	*
Endoplasmin	0.5164	**	0.4804	*
Isocitrate dehydrogenase [NADP], mitochondrial	0.5118	*	0.4164	*
Protein disulfide-isomerase A4	0.5076	*	0.4499	*
Protein ERGIC-53	0.4971	*	0.4479	*
Tubulin alpha-4A chain	0.4941	*	0.4951	*
Neutral alpha-glucosidase AB	0.483	*	0.396	ns
Guanine nucleotide-binding protein subunit beta-2-like	0.4502	*	0.3353	ns
Signal recognition particle subunit SRP72	0.4444	*	0.4308	*
Thioredoxin domain-containing protein 5	0.4049	*	0.4182	*
Poly [ADP-ribose] polymerase 1	0.3561	ns	0.2772	ns
Cartilage acidic protein 1	− 0.3355	ns	− 0.3434	ns
Heat shock 70 kDa protein 1A/1B (HSP70-1)	− 0.5366	**	− 0.5838	**
LEM domain-containing protein 2	− 0.6254	**	− 0.5722	**
Nesprin-3	− 0.6296	**	− 0.6015	**
Tenascin-X	− 0.6321	**	− 0.5679	**
Spectrin beta chain, non-eryththrocytic 1	− 0.6884	***	− 0.6734	***
Scavenger receptor class A member 5	− 0.7069	**	− 0.6619	**
Carboxypeptidase Q	− 0.7156	***	− 0.7019	**
Lamin-B2	− 0.7414	***	− 0.7293	***
Glutathione peroxidase 3	− 0.7475	***	− 0.7667	***
Beta-2-syntrophin	− 0.7522	***	− 0.8171	***
Creatine kinase B-type	− 0.7555	***	− 0.7136	***

Alarmins S100A8 and S100A9 were both correlated to the 50 other protein intensities obtained by MS/MS and correlated to the histological inflammatory score. r = coefficient correlation (Pearson test). *, **, *** Represent P-values < 0.05; 0.01 and 0.001, respectively.

*ns* not significant.

A special attention was drawn to the status of complement component protein levels: 18 have been identified among the 1871 proteins retained (Additional file 1). Only complement *C1QB* and *C1QBP* were slightly significantly correlated with the histological inflammatory score.

## Discussion

OA synovitis share at a lower degree many common features with RA synovitis including abnormal synoviocytes proliferation, leukocytes infiltration and angiogenesis. In our study, synovial hyperplasia and lymphocyte infiltration were observed in all samples. Macrophages infiltration was detected in all biopsies except for 2 OA and 1 CPPA synovitis. Plasmocytes infiltration was observed in all RA and in 33–43% of non-RA biopsies, whereas PMN infiltration was present in 75% of RA biopsies and in 0–10% of non-RA biopsies. The histological inflammatory score highlighted an inflammatory continuum through the 24 biopsies with an overlap between the three studied pathologies in agreement with other publications^1,2,15^. It emphasizes the absence of a unique pattern for each studied disease and the heterogeneity of cell infiltration, either quantitatively and qualitatively. Synovitis is composed of various inflammatory cells and proliferating synoviocytes that induce the secretion of classical inflammatory mediators but also the secretion of thousands of proteins that represents the dark proteome, a Gordian knot that can only be unraveled by a tissue proteomic analysis.

To the best of our knowledge, this is the first proteomic study of human synovitis for which proteins levels were compared to their corresponding histological inflammatory score. DAVID and STRING analyses highlighted 11 proteins involved in the endoplasmic reticulum pathway: *DNAJB11*, *CALR*, *ERP29*, *GANAB*, *HSP90B1*, *HSPA1A*, *HSPA5*, *HYOU1*, *LMAN1*, *PDIA4*, and *TXNDC5*. All these proteins were detected in the 24 biopsies supporting the relevance of their identifications. How can we connect ER proteins to the inflammatory process inside synovitis? There is a strong evidence of ER stress and inflammation cross talk^19^. In synovitis, inflammatory cells such as macrophages or neutrophils move through the synovial tissue to produce inflammatory cytokines and ROS. These factors can trigger ER stress and the UPR pathways. The NF-κB pathway plays a central role in the crosstalk between ER stress and inflammation by all three branches of the UPR^19^.

Endoplasmic reticulum (ER) is an intracellular organelle playing a major role in proper proteins folding through the activation of several chaperone proteins, including protein disulfide isomerase (PDI), ERP29, the Hsp70 family member Glucose-Regulated Protein 78 kDa (GRP78/BiP) (*HSPA5*), and calreticulin (*CALR*). However, despite the function of these chaperones, the success rate for proper folding is often quite low. Incompletely folded proteins are forced to be removed from cells by a process called UPR (unfolded protein response) activated along with the ER-associated degradation (ERAD), enhancing protein degradation by the proteasome. Some cellular disturbances such as nutrient deprivation, hypoxia or loss of calcium homeostasis can disrupt ER efficiency and lead to the accumulation of unfolded proteins enhancing a stress response in the ER.

Under normal conditions, GRP78/BiP (*HSPA5*) maintains the canonical UPR regulators (IRE1α, PERK and ATF6) in an inactivated form, while upon pathological conditions, it dissociates from the three UPR proteins inducing UPR activation^19^. Contribution of the ER stress in RA pathogenesis has been recently described^19^. Inflammation and ER stress work together by driving inflammatory cells to produce pro-inflammatory cytokines but also to enhance FLS proliferation and angiogenesis^19,20^. Further, synovial hyperplasia is linked to chronic inflammation and joint destruction^1^. GRP78/BiP has been localized predominantly in the lining but also sublining layers of RA (more pronounced) and OA synovium^20^. Down-regulation of GRP78/BiP increases apoptosis of RA FLS and conversely its overexpression prevents cells from apoptotic death induced by an ER stressor^20^. Selective abrogation of GRP78/BiP blunts activation of NF-*κ*B and protect mice from collagen arthritis^21^.

ERdj3 (*DNAJB11*) acts as a component with other co-chaperone proteins SDF2 and SDF2L1 in the GRP78/BiP chaperone cycle to prevent the aggregation of misfolded proteins^22^ and regulates GRP78/BiP occupancy in living cells^23^. Two other ER chaperones, such as GRP94/endoplasmin (*HSP90B1*) and calreticulin (*CALR*) contribute to the autoimmune process in different ways. Under physiologic conditions, GRP94/endoplasmin optimizes the function of B cells by chaperoning TLRs^24^. Indeed, GRP94/endoplasmin ablation in B cells attenuated antibody production in the context of TLR stimulation^24^. Under pathologic conditions, GRP94/endoplasmin translocates to the cell surface and extracellular space and could function as an autoantigen to induce autoantibodies and enhance immune responses^25^. GRP94/endoplasmin may also act as an endogenous ligand of TLR2 to promote chronic inflammation^25^. GRP94/endoplasmin induces the transcription of TLR2, TNF-α and IL8 but not TLR4 in synovial macrophages^26^. GRP94/endoplasmin is highly expressed in the lining and sublining layers of RA synovium correlating with lining thickness (lining) and the inflammatory score (sublining)^26^. Its expression was also detected in control OA synovium^26^. A recent study demonstrated that the upregulation of Bcl-XL and Mcl-1 expression in RA FLS by calreticulin promoted apoptosis resistance of RA FLS^27^. Calreticulin promotes angiogenesis by activating nitric oxide signaling pathway in RA^28^. Further, soluble calreticulin can induce the expression of pro-inflammatory cytokines by macrophages^29^. Calreticulin was previously detected by another proteomic study focusing on formalin-fixed paraffin-embedded (FFPE) synovial tissues provided from OA and RA tissues^12^.

Hypoxia-upregulated protein 1 (*HYOU1*) or GRP170 is co-regulated and associated with two other chaperones GRP78/BiP and GRP94/endoplasmin, suggesting their coordinated activity in the maintenance of protein homeostasis^30^. HYOU1 presents an important cytoprotective role in hypoxia-induced cellular perturbation^31^ and can contribute to cell survival when ER is under stress. However, surface or extracellular HYOU1 exerts documented immunoregulatory activities in some immunopathologies but its role in rheumatic diseases remains unknown^32^. In addition to its function as a “danger” molecule alerting the immune system of tissue damage, the extracellular HYOU1 has also the capacity of amplifying the inflammatory response triggered by microbial signals and possibly by DAMPs^32^. HYOU1 also promoted pulmonary fibrosis in mice by increasing pulmonary levels of TGF-β1 and myofibroblasts^33^.

Thioredoxin domain-containing protein 5 (*TXNDC5*) is a protein disulfide isomerase with clear pro-inflammatory properties. TXNDC5 contributes to abnormal RA FLS proliferation, migration and IL-6 production by inhibiting IGFBP1 expression^34^. Downregulation of TXNDC5 could contribute to RA FLS antiangiogenic and proapoptotic features through the suppression of CXCL10 and TRAIL^35^. Further, TXNDC5 synergizes with heat shock cognate 70 protein (HSC70) to exacerbate the inflammatory phenotype of RA FLS through NF-κB signaling^36^. TXNDC5 expression was increased in synovial tissues of RA patients compared to OA as identified by a proteomic study^13^ or by immunochemistry^37^. Further, elevated levels were found in the synovial fluid and serum of RA patients^37^.

The role of ERp29 (*ERP29*) seems controversial in the literature regarding to apoptosis. It protects cells such as neurons from apoptosis^38^ whereas it sensitizes some others such as cancer cells ^39^. Murine macrophages upon interaction with heat-inactivated Candida albicans unravel an anti-inflammatory response with the overexpression of ERp29^40^. Protein disulfide-isomerase-A4 (PDIA4) mediates resistance to cisplatin-induced cell death in lung adenocarcinoma^41^. PDIA4 mRNAs were significantly increased in patients' inflamed colonic mucosa compared to uninflamed mucosa and controls^42^. Protein ERGIC-53 (*LMAN1*) is a type I transmembrane protein that is located at the ER, ER-Golgi Intermediate Compartment (ERGIC) and cis-Golgi. Protein ERGIC-53 facilitates transport of several cargo proteins including factors critical to the coagulation cascade from the ER to Golgi (41). Interaction of Protein ERGIC-53 with β-amyloid protected cultured neuronal cells from β-amyloid-induced apoptosis^44^. GANAB is a key glycoprotein quality control protein in ER removing glucose residues from immature glycoproteins^45^. Although ERp29, PDIA4, Protein ERGIC-53 and GANAB proteins were highly positively correlated in our work with the histological inflammatory score, their presence and their role in the pathophysiology of synovitis have not yet been described.

As for other chaperones, the heat shock 70 kDa protein 1A/1B or Hsp72 (*HSPA1A*) can be released by normal cell under stress or by damaged cell, but unlike most of other chaperones, HSPA1A displays anti-inflammatory properties. Recombinant human HSPA1A suppresses the production of pro-inflammatory cytokines in RA FLS by inhibition of NF-κB pathway and decreases collagen-induced arthritis in mice^46^. Interestingly, in our study, HSPA1A levels were significantly lower in RA than in OA or CPPA synovium and negatively correlated with the histological inflammatory score as well as with the 10 other aforementioned protein levels of the ER pathway suggesting a defective anti-inflammatory response in favor of a pro-inflammatory one under the control of ER proteins.

S100A8 and S100A9 were expressed in the 24 biopsies and positively correlated with the histological inflammatory score. Further, they were almost correlated to all other 49 proteins in Table 2 highlighted for their correlation with the histological inflammatory score. S100A8 and S100A9 proteins are Ca2+ binding proteins constitutively expressed by neutrophils and monocytes. They are well-known DAMPs proteins participating to leukocyte recruitment and cytokines secretion and are highly expressed in many inflammatory conditions. They were detected in serum^47^, synovial fluid^48^ and joint tissue^12,13^ of RA patients. In the OA synovium, they are mainly produced by M-1 like macrophages and slightly by M-2 like macrophages but not by FLS, inducing inflammatory cytokines and MMPs expression^49^. S100A8 and S100A9 are actively involved in the thickening of the intimal layer and in the development of joint destruction in murine collagenase-induced OA but not in destabilized medial meniscus-induced OA^50^. Further, S100A8 and S100A9 levels in the OA synovitis significantly correlate with synovial lining thickness and cellularity in the subintima^50^. Gene and protein expression of S100A9 were increased in inflamed areas as compared to normal area of human OA synovitis^51^.

Eighteen complement component proteins were identified but none except complement C1QB (positively) and C1QBP (negatively) were correlated to the histological inflammatory score. These findings are in contrast with two other proteomic analyses^9,10^ performed in OA synovial fluids but not in tissue. Indeed, Gobezie et al*.* identified C3 as a discriminant marker exhibiting a sensitivity of 90% and a specificity of 85% in OA synovial fluids^9^. Similarly, we also identified previously increased levels of C3f. fragment in synovial fluid of OA patients^11^. Complement components can be delivered from blood by ultrafiltration. Numerous publications mentioned that complement components could be produced by synovial tissue cells^52^. However, in our study, complement component levels in OA, CPPA and RA were not correlated to the histological inflammatory score and were not statistically elevated among the three pathologies, suggesting that complement cascade was further playing a role in synovial fluids and not in synovial tissue.

## Conclusion

This study highlights the continuum existing between OA, CPPA and RA synovitis both at the protein and the histological inflammatory score levels. These two levels are connected giving pathophysiological relevance of the proteomic synovium analysis. Pannus development in these diseases is characterized by overexpression of many proteins involved in the ER stress, mostly chaperones and co-chaperones. All studied ER proteins except HSPA1A exhibit pro-inflammatory properties when they are exposed on the cell membrane or secreted outside favoring inflammatory cytokine production as well as proliferation and migration of FLS. Five proteins (*HSPA5, HSB90B1, CALR, TXNDC5* and *HSPA1A*) have been previously identified in the RA synovium and to a lesser extent in the OA synovium. Six proteins (*DNAJB11, HYOUI, ERp29, PDIA4, LMAN1 and GANAB*) have never been described in RA or in OA synovium, and none in CPPA synovium. These data confirm an important role for these chaperones and co-chaperones of the ER pathway in the pathophysiology of RA, but more importantly strongly suggest a similar unknown pattern in the pathophysiology of OA and CPPA. S100A8 and S100A9 were correlated to the histological inflammatory score and to most of highlighted proteins in Table 2. Complement components seem to behave independently from the histological inflammatory score. We have also highlighted the negative correlation between the histological inflammatory score and the HSPA1A/TXNDC5 ratio, which fits with the capacity of TXNDC5 to exacerbate the inflammatory phenotype of FLS^36^.

This proteomic study relies mainly on correlation observed between ER protein expression and the histological inflammatory score. It remains however necessary to confirm that ER proteins are causally or consequently related to inflammation in synovitis by other functional studies. This proteomic analysis suggests the need for future developments such as: (1) the identification of other pathways including other proteins that are not correlated with the histological inflammatory score, and (2) the characterization of protein clusters correlated with each type of cells infiltrating the pannus as well as with FLS proliferative capacity. This tool may allow to develop a molecular classification of complex rheumatic diseass.

## Supplementary information

Supplementary figure.

Supplementary information.

## Data Availability

The datasets used and/or analyzed during the current study are available from the corresponding author on reasonable request.

## Abbreviations

2D-nano-UPLC-ESI-Q-Orbitrap: 2 Dimensional-nano-ultra performance liquid chromatography-electrospay ionization-Q-Orbitrap
CALR: Calreticulin
CPPA: Chronic pyrophosphate arthropathy
CRP: C-reactive protein
DAMPS: Damage-associated molecular patterns
ER: Endoplasmic reticulum
ERAD: ER-associated degradation
FLS: Fibroblast-like synoviocytes
GRP78: Glucose-regulated protein 78 kDa
LC–MS/MS: Liquid chromatography–tandem mass spectrometry
LFQ: Label free quantification
MRI: Magnetic resonance imaging
OA: Osteoarthritis
PMN: Polymorphonuclear cells
PSM: Peptide spectrum match
RA: Rheumatoid arthritis
TLR: Toll-like receptors
TXNDC5: Thioredoxin domain-containing protein 5
UPR: Unfolded protein response
US: Ultrasonography
