# Supplementary figures and images for: Proteins involved in the endoplasmic reticulum stress are modulated in synovitis of osteoarthritis, chronic pyrophosphate arthropathy and rheumatoid arthritis, and correlate with the histological inflammatory score
Source: Sci Rep. 2020 Sep 4;10:14159. doi: 10.1038/s41598-020-70803-7 (PMC7473860; doi:10.1038/s41598-020-70803-7)

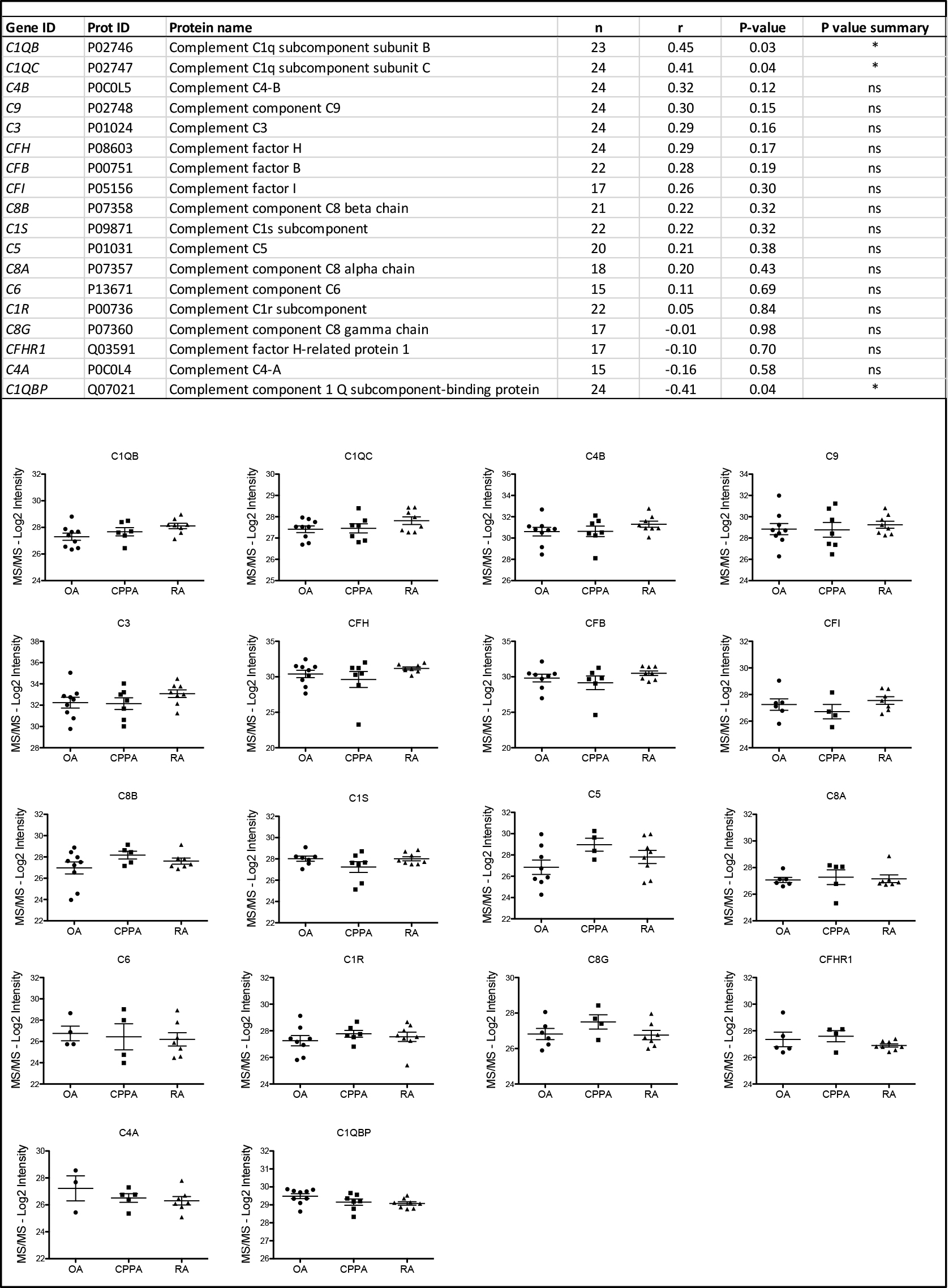

Supplement: Supplementary file 1 — Supplementary figure. [file 41598_2020_70803_MOESM1_ESM.jpg]
