# Supplementary material for: Proteins involved in the endoplasmic reticulum stress are modulated in synovitis of osteoarthritis, chronic pyrophosphate arthropathy and rheumatoid arthritis, and correlate with the histological inflammatory score
Source: Sci Rep. 2020 Sep 4;10:14159. doi: 10.1038/s41598-020-70803-7 (PMC7473860; doi:10.1038/s41598-020-70803-7)
Supplement: Supplementary file 2 — Supplementary information. [file 41598_2020_70803_MOESM2_ESM.docx]

**Supplementary information**

**Proteins involved in the endoplasmic reticulum stress are modulated in synovitis of osteoarthritis, chronic pyrophosphate arthropathy and rheumatoid arthritis, and correlate with the histological inflammatory score**

Dominique de Seny (1), Elettra Bianchi (2), Dominique Baiwir (3), Gaël Cobraiville (1), Charlotte Collin (1), Mégane Deliège (1), Marie-Joëlle Kaiser (1), Gabriel Mazzucchelli (4), Jean-Philippe Hauzeur (1), Philippe Delvenne (2), Michel G. Malaise (1).

**Affiliation:**

(1) Laboratory of Rheumatology, GIGA Research, CHU Liege, 4000 Liege, Belgium.

(2) Department of Pathology, GIGA Research, CHU Liege, 4000 Liège, Belgium

(3) GIGA Proteomics Facility, University of Liege, 4000 Liege, Belgium

(4) Mass Spectrometry Laboratory, MolSys Unit Research, University of Liege, 4000 Liege, Belgium

**ADDITIONAL FILE LEGEND**

**Additional file 1: Correlation parameters between complement components and the histological inflammatory score**

The table includes complement components listed according to their correlation coefficient with histological inflammatory score. Graphs represent the distribution of these complement components intensities obtained by MS/MS among the three groups (OA, CPPA and RA). * represent P-values < 0.05; ns = not significant; r= correlation coefficient.
